# Establishment of a pathomic-based machine learning model to predict CD276 (B7-H3) expression in colon cancer

**DOI:** 10.3389/fonc.2023.1232192

**Published:** 2024-01-08

**Authors:** Jia Li, Dongxu Wang, Chenxin Zhang

**Affiliations:** ^1^ Department of Gastroenterology, The 983rd Hospital of Joint Logistic Support Force of PLA, Tianjin, China; ^2^ Department of General Surgery, The 983rd Hospital of Joint Logistic Support Force of PLA, Tianjin, China

**Keywords:** CD276, pathomics, prognostic biomarkers, colon cancer, machine learning, histopathological images

## Abstract

CD276 is a promising prognostic indicator and an attractive therapeutic target in various malignancies. However, current methods for CD276 detection are time-consuming and expensive, limiting extensive studies and applications of CD276. We aimed to develop a pathomic model for CD276 prediction from H&E-stained pathological images, and explore the underlying mechanism of the pathomic features by associating the pathomic model with transcription profiles. A dataset of colon adenocarcinoma (COAD) patients was retrieved from the Cancer Genome Atlas (TCGA) database. The dataset was divided into the training and validation sets according to the ratio of 8:2 by a stratified sampling method. Using the gradient boosting machine (GBM) algorithm, we established a pathomic model to predict CD276 expression in COAD. Univariate and multivariate Cox regression analyses were conducted to assess the predictive performance of the pathomic model for overall survival in COAD. Gene Set Enrichment Analysis (GESA) was performed to explore the underlying biological mechanisms of the pathomic model. The pathomic model formed by three pathomic features for CD276 prediction showed an area under the curve (AUC) of 0.833 (95%CI: 0.784-0.882) in the training set and 0.758 (95%CI: 0.637-0.878) in the validation set, respectively. The calibration curves and Hosmer-Lemeshow goodness of fit test showed that the prediction probability of high/low expression of CD276 was in favorable agreement with the real situation in both the training and validation sets (*P*=0.176 and 0.255, respectively). The DCA curves suggested that the pathomic model acquired high clinical benefit. All the subjects were categorized into high pathomic score (PS) (PS-H) and low PS (PS-L) groups according to the cutoff value of PS. Univariate and multivariate Cox regression analysis indicated that PS was a risk factor for overall survival in COAD. Furthermore, through GESA analysis, we found several immune and inflammatory-related pathways and genes were associated with the pathomic model. We constructed a pathomics-based machine learning model for CD276 prediction directly from H&E-stained images in COAD. Through integrated analysis of the pathomic model and transcriptomics, the interpretability of the pathomic model provide a theoretical basis for further hypothesis and experimental research.

## 1 Introduction

Colon cancer is the most common malignancy of the digestive system in humans (1). Despite advances in surgery and chemotherapy, recurrence and death rates of colon cancer have not decreased significantly in recent decades (2). Traditional prognostic indicators of colon cancer, mainly TNM staging, fail to meet the clinical needs of precision medicine (3, 4). There is an urgent need to explore novel prognostic indicators to stratify patients and provide guidance for individualized precision therapy. Besides, immunotherapy is one of the promising therapeutic options for malignancy developed in recent years (5). Currently, the effectiveness of inhibitors targeting immune checkpoints, primarily CTLA-4 and PD-1/PD-L1, have been demonstrated in multiple clinical studies (6, 7), making immune checkpoint molecules to be the focus of current research (8). However, many patients show no response or resistance to these immune checkpoint inhibitors, so other potential immune checkpoint targets need to be explored.

CD276 (B7-H3), a member of the B7 family of immune checkpoint proteins, have been found to be low expressed in normal tissues and high expressed in a variety of malignancies, including colorectal cancer (9–11). Emerging evidence shows that CD276 plays a key role in tumor progression, treatment resistance and poor prognosis (12). Furthermore, a study on MGC018, an antibody-drug conjugate targeting CD276, showed effective antitumor activity in a variety of human tumor xenografts such as breast and lung cancers and favorable safety in animal models. The efficacy and safety demonstrated in this study support sustained study on MGC018 for the therapy of tumors (13). Accordingly, the exploration on the role of CD276 in colon cancer has dual potential of prognostic prediction and therapeutic target for clinical application.

At present, the existing CD276 testing methods cannot be widely promoted in clinical practice, because the genetic test such as qPCR or RNA sequencing requires additional fresh tissue samples and immunohistochemical staining is expensive and time-consuming. Therefore, it is both promising and challenging to explore a universally applicable method for CD276 detection in cancer population.

H&E-stained sections are the most accessible image data necessary for clinical diagnosis. Compared with immunohistochemistry, H&E staining is more robust, efficient, and inexpensive without the influence of antibodies. However, pathologists cannot predict the expression of CD276 or other biomarkers from the H&E-stained images. With the development of artificial intelligence (AI), pathomics comes into being (14, 15). Pathomics contains enormous amount of data of quantitative features transformed from histopathological images by AI, and these data show a broad application prospect in diagnosis, molecular expression and prognosis prediction (14, 16). A recent study reported histopathological features from H&E staining images for microsatellite instability prediction in colorectal cancer (17), and some biomarkers in various cancers likewise (5, 18). However, there is no evidence that H&E-stained images can predict CD276 expression in colon cancer.

In this study, we developed a gradient boosting machine (GBM)-based pathomic model that could predict CD276 expression from H&E-stained pathological images. On account of CD276 has just been identified as a potential biomarker in colon cancer recently and has not yet become part of routine clinical application, there is certain difficulty to establish a large database that integrate CD276 expression and H&E-stained images. In this study, we collected a dataset of colon adenocarcinoma (COAD) patients with H&E staining and transcriptome sequencing including CD276 from the Cancer Genome Atlas (TCGA) database. Furthermore, we illustrated the underlying mechanism of the pathomic features by associating the pathomic model with transcription profiles.

## 2 Materials and methods

### 2.1 Study cohort

**Figure 1:**
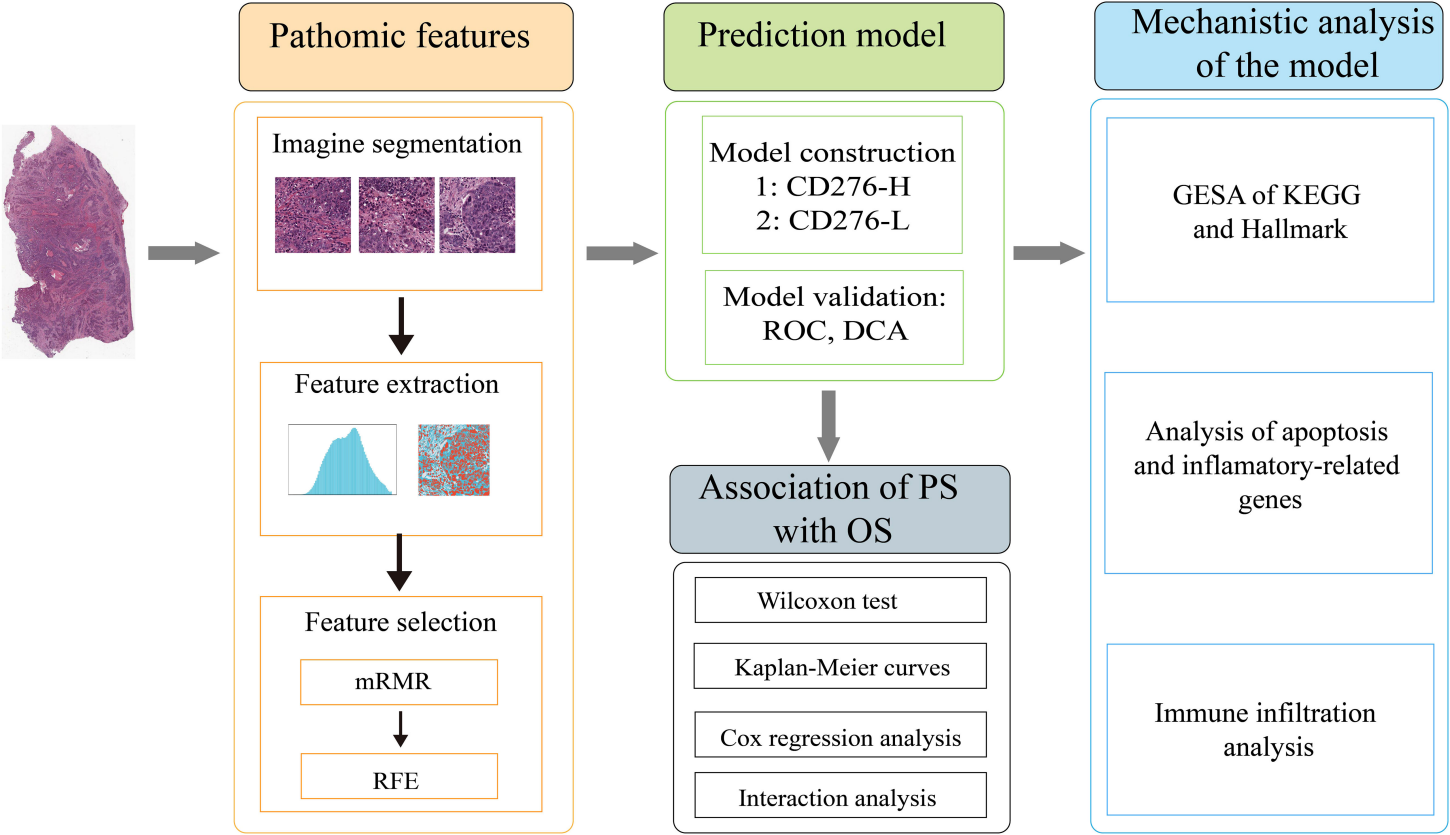
An overview of the study design.

A dataset of COAD patients with clinical parameters, RNA sequencing information, and complete H&E-stained histopathological images was retrieved from TCGA database. Inclusion criteria: patients with pathologically diagnosed colon adenocarcinoma, RNA sequencing information and naïve treatment. Exclusion criteria: (a) patients with missing survival data or survival time less than one month; (b) patients without clinical parameters of tumor stage or grade; (c) patients with unqualified pathological images. The H&E-stained histopathological images in svs format with a maximum magnification of 20× or 40× were downloaded from TCGA database (19, 20). An overview of the study design was shown in **Figure 1**. The RNA sequencing data for normal tissues, specifically non-tumoral colon tissues from colon adenocarcinoma patients, were also sourced from the TCGA database.

Demographic and clinical parameters included in the study contained age (<65 or ≥65 years), gender (female or male), pathological stage (I/II or III/IV), colonic polyps (no, unknown, or yes), history of colonic polyps (no, unknown, or yes), lymph node metastasis (no, unknown, or yes), perineural invasion (no, unknown, or yes), venous invasion (no, unknown, or yes), pathological type (colon adenocarcinoma or colon mucinous adenocarcinoma), residual tumor (R0, R1/R2, or Rx/Unkown), tumor status (tumor free, unknown, or with tumor), tumor location (left or right), and chemotherapy (no or yes).

### 2.2 Evaluation of CD276 expression

To perform the differential expression analysis of CD276 between tumor tissue and normal tissue, we acquired and meticulously processed RNA sequencing data from the TCGA database for the TCGA-COAD project. This data was processed using the STAR workflow, and we extracted TPM-formatted data for this specific analysis. For the prognostic analysis of CD276, we conducted mRNA-seq data analysis utilizing FPKM-formatted data. To ensure uniformity in the FPKM/TPM values across the RNA sequencing data, we applied FPKM/TPM-log_ratio standardization, as referenced in previous studies (21), for subsequent analysis. The expression levels of CD276 in COAD samples were compared with those in normal colon samples by Wilcoxon rank sum test. Drawing upon previous studies (22–24), the “surv_cutpoint” function in the “Survminer” package of R was used to calculate the cutoff value of CD276 expression for all patients based on a minimum p-value method. Based on this determined cutoff value, all patients were then categorized into two groups: the high CD276 expression (CD276-H) group and the low CD276 expression (CD276-L) group. The Kaplan-Meier (KM) curves were used to show the effects of demographic and clinical parameters, including CD276, on overall survival (OS), which was represented by median survival time. Log-rank test was used to assess the differences in OS among the groups. Univariate Cox regression analysis was used to evaluate the relationship between each parameter and OS of COAD, and then all variables were included in multivariate analysis to explore whether each parameter was an independent factor affecting OS of COAD when adjusting recognized confounders. Stratified univariate Cox regression analysis of CD276 expression (high/low) and OS of COAD was performed to assess the interactions between CD276 and covariates.

The original dataset was divided into the training set and the validation set according to the ratio of 8:2 using a stratified sampling method, so as to ensure that the proportion of patients with CD276 high to low expression was equal in the two sets.

### 2.3 Preprocessing and segmentation of histopathological images

For H&E-stained pathological images, the background was removed using OTSU algorithm and the tissue foreground for study were obtained (25, 26). The 40× images were divided into multiple sub-images with 1024×1024 pixel. The 20× images were divided into multiple sub-images with 512×512 pixel, which were subsequently resized to a resolution of 1024×1024 pixel. Sub-images were reviewed by two experienced pathologists to exclude those with poor quality, such as contaminated or blurred images or images with more than 50% blank areas. For each patient, 10 sub-images were randomly selected for subsequent analysis (19).

### 2.4 Feature extraction of histopathological images and data preprocessing

“PyRadiomics” package (https://pyradiomics.readthedocs.io/en/latest/) was used to extract features including original features and higher-order features of each sub-image. For each patient, ten sub-images were randomly chosen to calculate image features, and then the average value of each feature was taken for subsequent data analysis (27).

“Caret” package was used to conduct z-score standardization of the features in the training set to eliminate the difference degree between values of features. Then the mean and standard deviation of the training set were used to standardize the data of the validation set.

### 2.5 Feature selection and model construction and assessment

In order to eliminate redundancy between pathological features and avoid overfitting of the model, the Maximum relevance and minimum redundancy (mRMR) algorithm and the Recursive feature elimination (RFE) algorithm, using “mRMRe” and “caret” packages of R respectively, were successively applied to select the optimal feature subsets. Specifically, the mRMR was applied to screen a subset of features that had the greatest correlation with the biomarker to be predicted and the least correlation between the features. Then, the RFE with ten-fold cross validation was used to rank the importance of the selected features, and features of little importance are excluded in sequence. Finally, an accurate subset of predictive features was identified.

The Gradient Boosting Machine (GBM) method was employed to develop a pathomic model predicting the expression of CD276 based on the selected pathological features (28, 29). Receiver operating characteristic curves (ROC), Calibration curves, and decision curve analysis (DCA) curves were plotted through pROC, rms, and rmda packages respectively to assess the predictive efficacy, calibration, and clinical benefit of the model.

### 2.6 Association of the pathomic score with prognosis in COAD

The pathomic score (PS) of each patient was calculated according to the pathomic model. The “surv_cutpoint” function in the “Survminer” package of R was used to analyze the cutoff value of PS, according to which the subjects in both the training and validation sets were classified into high PS (PS-H) group and low PS (PS-L) group. Kaplan-Meier survival curve and Cox regression analysis were performed to evaluate the association of PS and OS in COAD. Univariate Cox regression analysis was use to evaluate the relationship between PS (high/low) and OS of COAD, and then multivariate analysis was used to explore independent factors affecting OS of COAD. Stratified univariate Cox regression analysis of PS high/low and OS of COAD was performed to assess the interactions between PS and covariates.

### 2.7 Analysis of biological significance of the pathomic model

Gene Set Enrichment Analysis (GSEA) based on Kyoto Encyclopedia of Genes and Genomes (KEGG) (c2.cp.kegg.v7.5.1.symbols.gmt) and Hallmark (hall.v7.5.1.symbols.gmt) were performed by “clusterProfiler” package of R. The ranking method employed in GSEA was primarily founded on gene expression levels. The top 10 pathways were visualized derived from both KEGG and Hallmark gene sets. The lists of genes associated with inflammatory response and apoptosis were downloaded from the KEGG and hallmark gen sets, respectively (30–32). The abundances of these genes were compared by Wilcoxon rank sum test to assess PS-related perturbations. The gene expression matrix of all patients was uploaded to the ImmuCellAI database (http://bioinfo.life.hust.edu.cn/ImmuCellAI/#!/) to calculate the immune cells of each patient.

### 2.8 Nomogram development

Clinical variables were screened using the Akaike information criterion (AIC), and the selected clinical variables were then integrated with pathological scores to build a nomogram model. This model was designed to predict the survival probability of COAD patients at 12, 24, and 36 months.

### 2.9 Statistical analysis

Categorical data were expressed by numbers (percentages) and differences between groups were calculated by Chi-square test. Continuous data were expressed by medians (Q1, Q3) and differences between groups were analyzed using Wilcoxon rank-sum test. Log-rank test was used to compare the survival rates between groups. Hosmer-Lemeshow test was performed to evaluate the calibration of the pathomic model using ResourceSelection package. A two-tailed *P*<0.05 was considered statistically significant. All statistical analysis and visualization were performed through R package (Version 4.1.0).

## 3 Results

### 3.1 Subject characteristics

A total of 332 COAD patients were included in this study from the TCGA database. According to the cut-off value of CD276 expression as 4.354 calculate by the “Survminer” package, all patients were divided into the CD276-H group (n=133) and the CD276-L group (n=199). As show in **Table 1**, there were no significant differences in demographic data and clinical characteristics between the CD276-H and CD276-L groups.

**Table 1:** Characteristics of subjects in the CD276-H and CD276-L groups.

Characteristics	Total (n=332)	CD276-L group (n=199)	CD276-H group (n=133)	*P* value
Age (years), n (%)				0.316
<65	132 (40)	84 (42)	48 (36)	
≥65	200 (60)	115 (58)	85 (64)	
Gender, n (%)				0.226
Female	160 (48)	90 (45)	70 (53)	
Male	172 (52)	109 (55)	63 (47)	
Pathological stage, n (%)				0.961
I/II	189 (57)	114 (57)	75 (56)	
III/IV	143 (43)	85 (43)	58 (44)	
Colonic polyps, n (%)				0.66
No	111 (33)	64 (32)	47 (35)	
Unknown	164 (49)	98 (49)	66 (50)	
Yes	57 (17)	37 (19)	20 (15)	
History of colonic polyps, n (%)				0.059
No	184 (55)	119 (60)	65 (49)	
Unknown	45 (14)	28 (14)	17 (13)	
Yes	103 (31)	52 (26)	51 (38)	
Lymph node metastasis, n (%)				0.283
No	180 (54)	114 (57)	66 (50)	
Unknown	22 (7)	14 (7)	8 (6)	
Yes	130 (39)	71 (36)	59 (44)	
Perineural invasion, n (%)				0.109
No	105 (32)	70 (35)	35 (26)	
Unknown	195 (59)	114 (57)	81 (61)	
Yes	32 (10)	15 (8)	17 (13)	
Venous invasion, n (%)				0.337
No	220 (66)	138 (69)	82 (62)	
Unknown	36 (11)	19 (10)	17 (13)	
Yes	76 (22)	42 (21)	34 (26)	
Pathological type, n (%)				0.133
Colon adenocarcinoma	285 (86)	176 (88)	109 (82)	
Colon mucinous adenocarcinoma	47 (14)	23 (12)	24 (18)	
Residual tumor, n (%)				0.746
R0	254 (77)	151 (76)	103 (77)	
R1/R2	20 (6)	11 (6)	9 (7)	
Rx/Unkown	58 (17)	37 (19)	21 (16)	
Tumor status, n (%)				0.284
Tumor free	153 (46)	94 (47)	59 (44)	
Unknown	28 (8)	20 (10)	8 (6)	
With tumor	151 (45)	85 (43)	66 (50)	
Tumor location, n (%)				1
Left	133 (40)	80 (40)	53 (40)	
Right	199 (60)	119 (60)	80 (60)	
Chemotherapy, n (%)				0.75
No	210 (63)	124 (62)	86 (65)	
Yes	122 (37)	75 (38)	47 (35)	

### 3.2 Clinical consequence of CD276 in COAD

**Figure 2:**
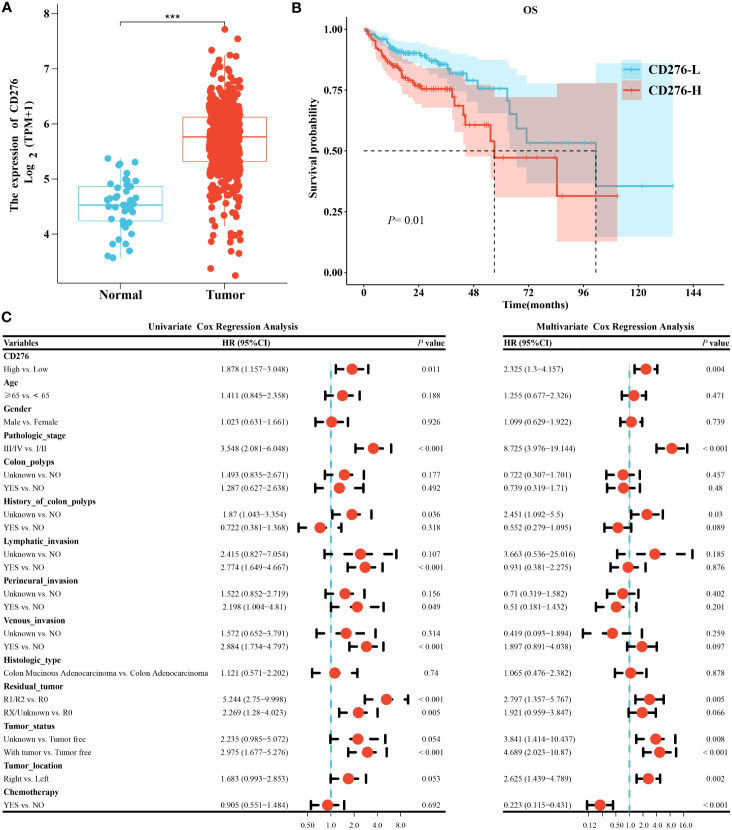
Analysis of clinical consequence of CD276 in COAD. **(A)** The expression level of CD276 in COAD tissues was significantly higher than that in normal colon tissues (n=41). **(B)** The Kaplan-Meier curve indicated that OS in the CD276-H group was significantly worse than that in the CD276-L groups. **(C)** Univariate and multivariate Cox regression analyses revealed that high expression of CD276 was an independent risk factor for OS of COAD. ***P<0.001.

The expression of CD276 in COAD samples was significantly elevated than that in normal colon tissues (n=41) (*P<*0.001) (**Figure 2A**). Kaplan-Meier survival curve showed that the median survival time of the CD276-L group was 101.4 months, and that of the CD276-H group was 57.5 months. Log-rank test showed that the OS of the CD276-H group was significantly poorer than that of the CD276-L group (*P=*0.01) (**Figure 2B**), suggesting that high expression of CD276 was significantly associated with poor prognosis of COAD.

To further clarify the effect of CD276 expression (high/low) on the prognosis of COAD, Cox regression analysis was performed. Univariate analysis showed that high expression of CD276 was a risk factor for OS (HR=1.878, 95% CI: 1.157−3.048, *P=*0.011) (**Figure 2C**). Furthermore, multivariate analysis indicated that high expression of CD276 was an independent risk factor for OS (HR=2.325, 95% CI: 1.3−4.157, *P=*0.004) (**Figure 2C**).

The HR between CD276 expression (high/low) and OS of COAD were not significantly different between patients aged <65 years (HR=1.443, 95% CI: 0.62-3.357, *P=*0.39) and those aged ≥65 years (HR=2.149, 95% CI: 1.185-3.896, *P=*0.012) (*P* =0.43 for the interaction), showing age does not play an interactive role in the association of CD276 expression and OS of COAD (**Supplementary Figure 1**). Similar effects were seen in subgroup comparisons according to other parameters of demographic and clinical characteristics (all *P>*0.05 for the interaction) (**Supplementary Figure 1**).

### 3.3 Development and performance assessment of the pathomic model

A stratified sampling method was used to divide all patients into the training set (n=267) and the validation set (n=65) in a ratio of 8:2. As listed in **Table 2**, no significant differences were found in demographic data and clinical characteristics between the two sets.

**Table 2:** Characteristics of subjects in the training and validation sets.

Characteristics	Total (n=332)	Training set (n=267)	Validation set (n=65)	*P* value
CD276, n (%)				1
Low	199 (60)	160 (60)	39 (60)	
High	133 (40)	107 (40)	26 (40)	
Age (years), n (%)				0.704
<65	132 (40)	108 (40)	24 (37)	
≥65	200 (60)	159 (60)	41 (63)	
Gender, n (%)				1
Female	160 (48)	129 (48)	31 (48)	
Male	172 (52)	138 (52)	34 (52)	
Pathological stage, n (%)				0.209
I/II	189 (57)	147 (55)	42 (65)	
III/IV	143 (43)	120 (45)	23 (35)	
Colonic polyps, n (%)				0.725
No	111 (33)	92 (34)	19 (29)	
Unknown	164 (49)	130 (49)	34 (52)	
Yes	57 (17)	45 (17)	12 (18)	
History of colonic polyps, n (%)				0.479
No	184 (55)	152 (57)	32 (49)	
Unknown	45 (14)	36 (13)	9 (14)	
Yes	103 (31)	79 (30)	24 (37)	
Lymph node metastasis, n (%)				0.236
No	180 (54)	149 (56)	31 (48)	
Unknown	22 (7)	15 (6)	7 (11)	
Yes	130 (39)	103 (39)	27 (42)	
Perineural invasion, n (%)				0.303
No	105 (32)	84 (31)	21 (32)	
Unknown	195 (59)	154 (58)	41 (63)	
Yes	32 (10)	29 (11)	3 (5)	
Venous invasion, n (%)				0.018
No	220 (66)	186 (70)	34 (52)	
Unknown	36 (11)	28 (10)	8 (12)	
Yes	76 (22)	53 (20)	23 (35)	
Pathological type, n (%)				0.062
Colon adenocarcinoma	285 (86)	224 (84)	61 (94)	
Colon mucinous adenocarcinoma	47 (14)	43 (16)	4 (6)	
Residual tumor, n (%)				0.294
R0	254 (77)	209 (78)	45 (69)	
R1/R2	20 (6)	15 (6)	5 (8)	
Rx/Unkown	58 (17)	43 (16)	15 (23)	
Tumor status, n (%)				0.946
Tumor free	153 (46)	122 (46)	31 (48)	
Unknown	28 (8)	23 (9)	5 (8)	
With tumor	151 (45)	122 (46)	29 (45)	
Tumor location, n (%)				0.329
Left	133 (40)	103 (39)	30 (46)	
Right	199 (60)	164 (61)	35 (54)	
Chemotherapy, n (%)				0.122
No	210 (63)	163 (61)	47 (72)	
Yes	122 (37)	104 (39)	18 (28)	
OS, n (%)				0.584
Alive	266 (80)	216 (81)	50 (77)	
Dead	66 (20)	51 (19)	15 (23)	
OS time (months), median (Q1, Q3)	22.32 (13.19, 35.48)	21.67 (13.18, 34.48)	24.73 (14.13, 39.53)	0.23

Q1, First Quartile; Q3, Third Quartile.

**Figure 3:**
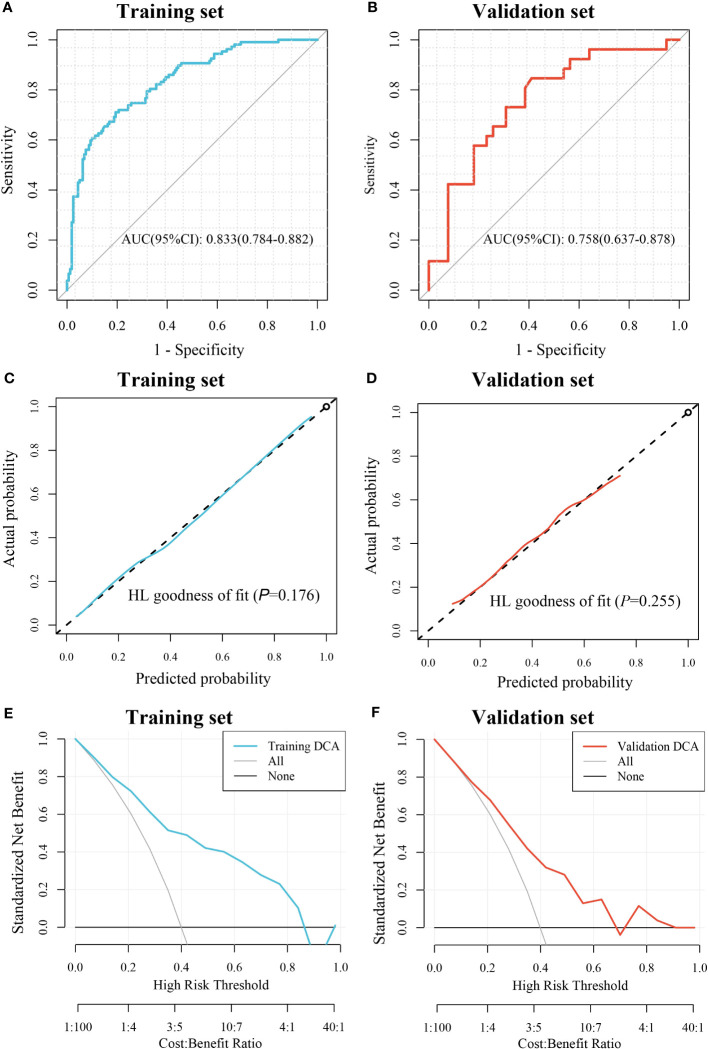
Evaluation of the pathomic model for prediction of CD276 expression. **(A)** ROC curve for the performance of the model in training set. **(B)** ROC curve for the performance of the model in the validation set. **(C)** Calibration curve of the model in the training set. **(D)** Calibration curve of the model in the validation set; **(E)** DCA of the model in the training set. **(F)** DCA of the model in the validation set.

A total of 1488 quantitative histopathological features were extracted. Twenty features were retained after filtering by the mRMR algorithm, and then three features, including lbp_2D_gldm_SmallDependenceEmphasis, wavelet_HH_firstorder_Mean, and original_firstorder_Mean were retained after screening by the RFE algorithm (**Supplementary Figure 2A**). The GBM algorithm was used to construct a pathomic model for CD276 prediction based on these three features in the training set. The importance of these features in the model was shown in **Supplementary Figure 2B**. The pathomic model showed AUC performance for the prediction of CD276 expression was 0.833 (95% CI: 0.784-0.882) in the training set and 0.758 (95% CI: 0.637-0.878) in the validation set, respectively (**Figures 3A, B**). In the training set, the accuracy was 0.768, the sensitivity was 0.71, the specificity was 0.806, and the Brier score was 0.178, and these indexes in the validation set were 0.692, 0.692, 0.692, and 0.201, respectively. The calibration curves and Hosmer-Lemeshow goodness of fit test showed that the prediction probability of CD276 expression was in favorable agreement with the real situation in both the training and validation sets (*P=*0.176 and 0.255, respectively) (**Figures 3C, D**). The DCA curves indicated that the pathomic model acquired high clinical benefit (**Figures 3E, F**).

### 3.4 Prognostic significance of the pathomic model in COAD

**Figure 4:**
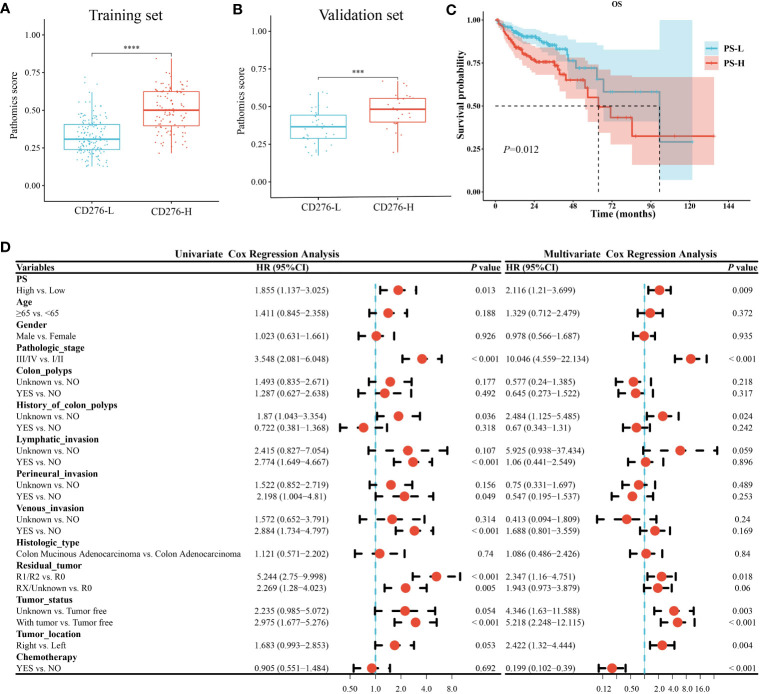
Analysis of clinical consequence of PS in COAD. A-B. PS of the CD276-H group was significantly higher than that of the CD276-L group in both the training set (*P*<0.05) **(A)** and the validation set (*P*<0.05) **(B)**. **(C)** Kaplan-Meier curve showed the OS in PS-H group was significantly worse than that in PS-L group (*P*=0.01). **(D)** Univariate and multivariate Cox regression analyses revealed that high PS was an independent risk factor for OS of COAD. ***P<0.001 and ****P<0.0001.

PS of the CD276-H group was significantly higher than that of the CD276-L group in the training set (*P<*0.05) (**Figure 4A**), as well as in the validation set (*P<*0.05) (**Figure 4B**). Using the “survminer” package, the cutoff value of PS as 0.4206 was obtained. Accordingly, all patients were divided into the PS-H group (n=140) and the PS-L group (n=192). As shown in **Supplementary Table 1**, the demographic data and clinical parameters were comparable between the PS-H and PS-L groups (*P>*0.05).

Kaplan-Meier survival curve showed that the median survival time in the PS-L group was 101.4 months, and that in the PS-H group was 63.67 months. Log-rank test showed that the OS in the PS-H group was significantly worse than that in the PS-L group (*P=*0.01) (**Figure 4C**), suggesting that high PS was significantly associated with poor prognosis of COAD.

Univariate and multivariate Cox regression analysis both showed that high PS was a risk factor for OS in COAD [(HR=1.855, 95% CI: 1.137-3.025, *P=*0.013) and (HR=2.116, 95% CI: 1.21-3.699, *P=*0.009), respectively] (**Figure 4D**). Stratified analysis showed that the HR between PS and OS of COAD were not significantly different between patients aged <65 years (HR=1.979, 95% CI: 0.849-4.613, *P=*0.11) and those aged ≥65 years (HR=1.903, 95% CI: 1.044-3.47, *P=*0.036) (*P*=0.94 for the interaction), showing age did not play an interactive role in the association between PS and OS of COAD. Similar effects were seen in subgroup comparisons according to other parameters of demographic and clinical characteristics (all *P>*0.05 for the interaction) (**Supplementary Figure 3**). The abovementioned stratified results suggested that PS was independently associated with the prognosis of COAD.

### 3.5 GSEA indicates several possible biological processes underlying the pathomic model

**Figure 5:**
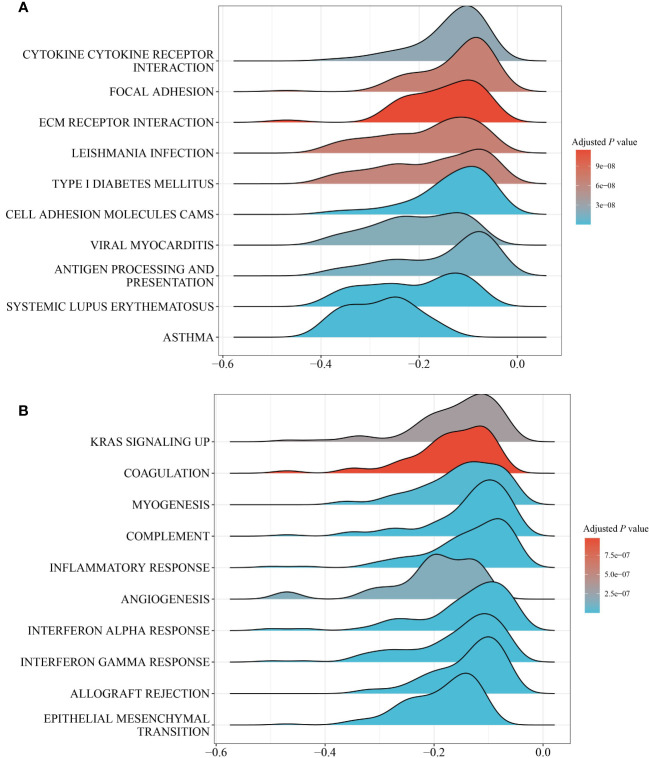
GESA of pathways between the PS-H and PS-L groups. **(A)** Top 10 KEGG pathways. **(B)** Top 10 hallmark pathways.

GSEA was performed between the PS-H and PS-L groups to explore the potential biological mechanism of the pathomic model. KEGG analysis identified that the top 10 pathways associated with PS were Cytokine-cytokine receptor interaction, focal adhesion, ECM receptor interaction, Leishmania infection, Type 1 diabetes mellitus, Cell adhesion molecules (CAMs), Viral myocarditis, Antigen processing and presentation, Systemic lupus erythematosus, and Asthma (**Figure 5A**). Hallmark analysis revealed that the top 10 pathways related to PS were inflammatory response, kras signaling up, coagulation, myogenesis, complement, angiogenesis, interferon-alpha response, interferon-gamma response, allograft rejection, and epithelial mesenchymal transition (**Figure 5B**).

### 3.6 Alterations in inflammatory response-related genes associated with PS

**Figure 6:**
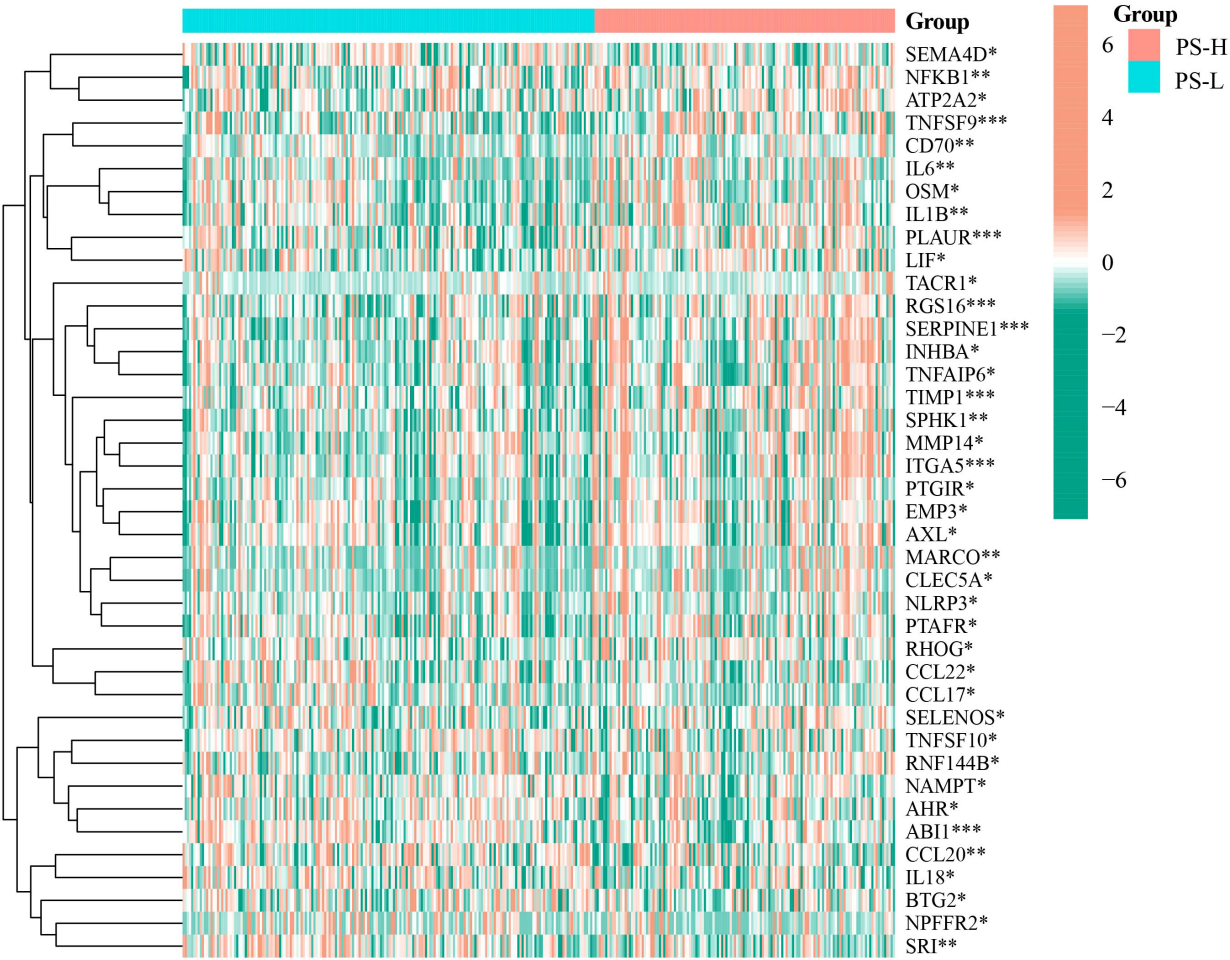
Differential expression analysis of inflammatory response-related genes in the PS-H and PS-L groups. The color bar represents log2(FPKM+1) of each gene. *P<0.05, **P<0.01, and ***P<0.001.

Wilcoxon rank-sum test showed that PLAUR, MARCO, OSM, TIMP1, SERPINE1, NFKB1, TACR1, INHBA, TNFAIP6, IL1B, TNFSF9, CD70, LIF, SELENOS, IL6, RNF144B, EMP3, RGS16, MMP14, PTGIR, ITGA5, NLRP3, AXL, PTAFR, ATP2A2, SPHK1, RHOG and CLEC5A were enriched in the PS-H group (*P<*0.05), while NPFFR2, SRI, CCL22, CCL17, NAMPT, AHR, CCL20, TNFSF10, ABI1, IL18, BTG2 and SEMA4D were enriched in PS-L group (*P<*0.05) (**Figure 6**).

### 3.7 Changes in apoptosis-related genes associated with PS

**Figure 7:**
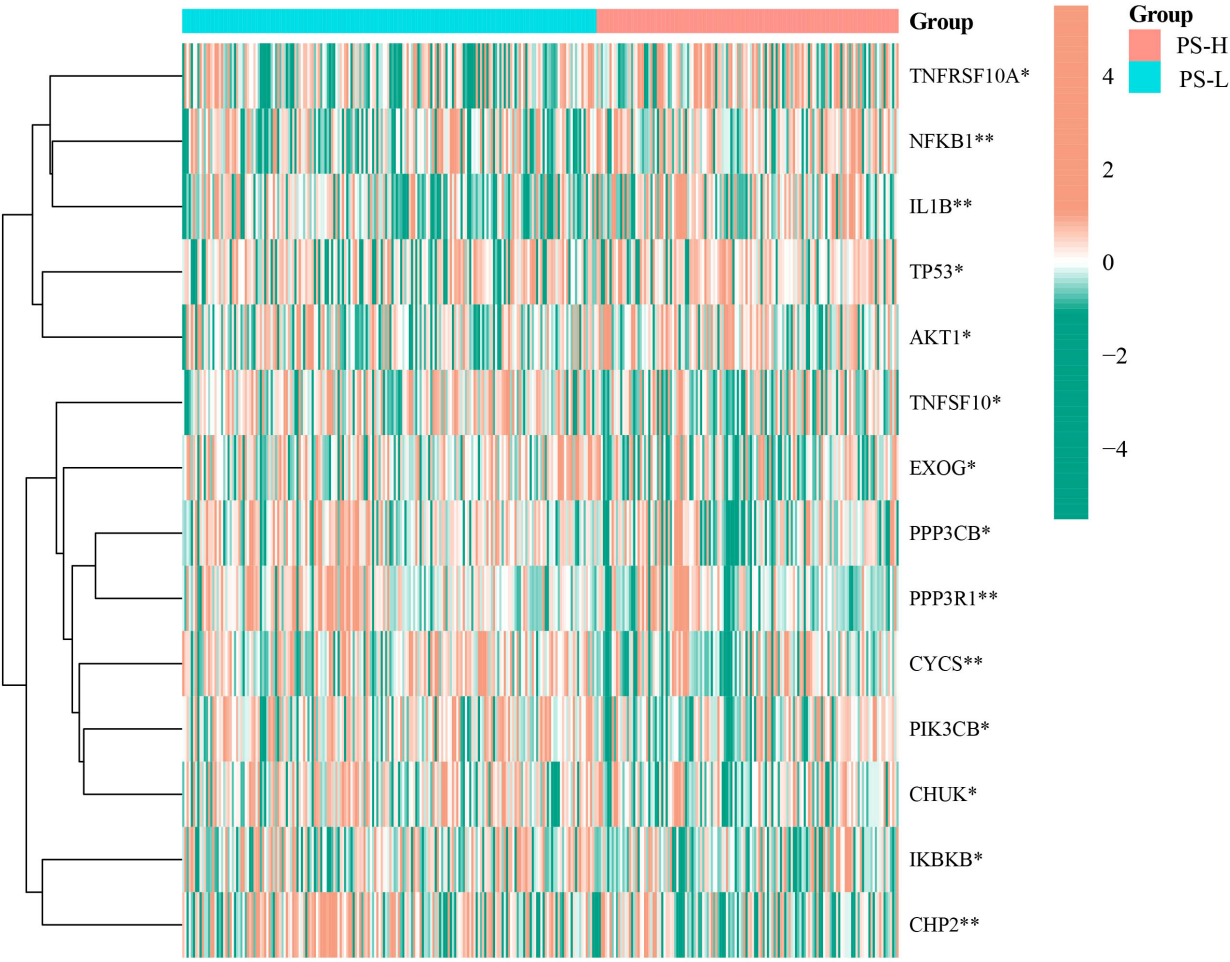
Differential expression analysis of apoptosis-related genes in the PS-H and PS-L groups. The color bar represents log2(FPKM+1) of each gene. *P<0.05 and **P<0.01.

Compared with the PS-L group, the expressions of TNFSF10A, NFKB1, IL1B, TP53, and AKT1 were significantly elevated (*P<*0.05), while that of PIK3CB, IKBKB, PPP3CB, TNFFS10, EXOG, CHP2, CYCS, CHUK, and PPP3R1 were significantly decreased in the PS-H group (*P<*0.05) (**Figure 7**).

### 3.8 Differences in immune cells associated with PS

**Figure 8:**
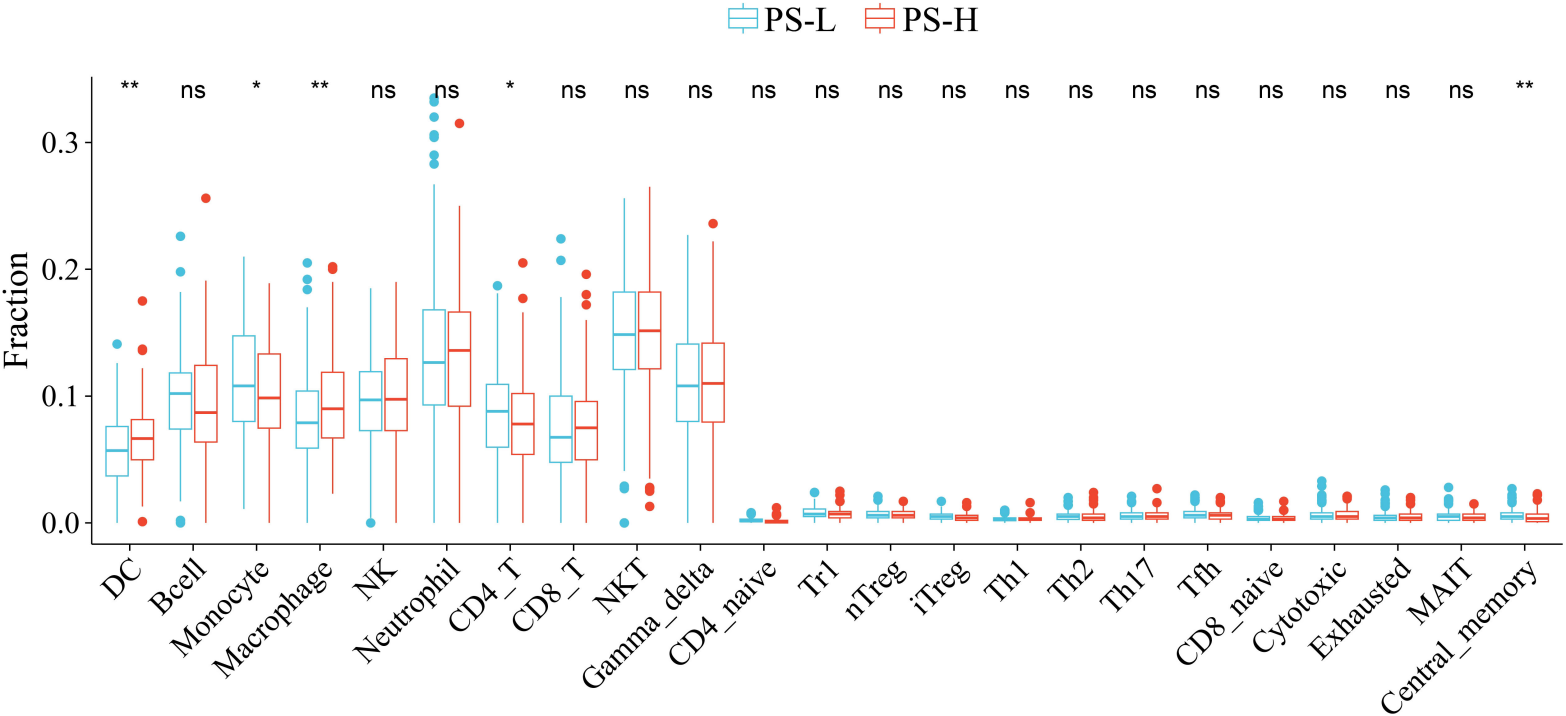
Comparison of the abundances of immune cells between the PS-H and PS-L groups. *P<0.05 and **P<0.01. ns, not significant.

A total of 5 types of immune cells were found to be significantly different between the PS-H and PS-L groups. Specifically, the abundances of DC and Macrophage were significantly higher in PS-H group, while those of Monocyte, CD4+ T cells and central memory were significantly enriched in the PS-L group (*P<*0.05) (**Figure 8**).

### 3.9 Development and validation of the nomogram for prognosis in COAD

**Figure 9:**
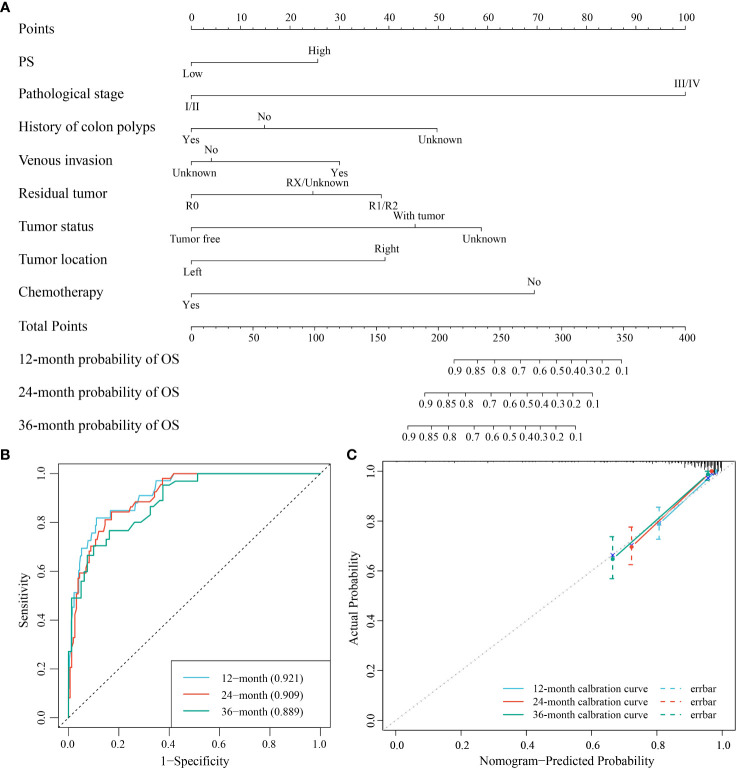
The nomogram model for the prediction of the survival probability of COAD patients at 12, 24, and 36 months. **(A)** The nomogram model for the survival probability. **(B)** ROC curve for the performance of the model. **(C)** Calibration curve of the model.

Using AIC criteria, we developed a nomogram model incorporating pathologic stage, history of colon polyps, venous invasion, residual tumor, tumor status, tumor location, chemotherapy, and PS to predict the survival probability of COAD patients at 12, 24, and 36 months (**Figure 9A**). The nomogram model demonstrated excellent discrimination with area under the curve (AUC) values of 0.921 (95% CI: 0.88-0.963), 0.909 (95% CI: 0.866-0.952), and 0.889 (95% CI: 0.832-0.947) for predicting survival probabilities at respective time points of interest (**Figure 9B**). The calibration curves and Hosmer-Lemeshow goodness-of-fit test confirmed that our prediction probabilities aligned well with actual outcomes for COAD patients at each time point assessed (**Figure 9C**). Furthermore, DCA curves indicated that our nomogram model provided good clinical benefit (**Supplementary Figures 4A-C**).

## 4 Discussion

The detection of CD276 may provide considerable information for the immunology and prognosis in various tumors. Nevertheless, its general application in clinical practice is limited due to the need for extra immunohistochemical or genetic testing. In this study, we established a pathomic model by machine learning method directly from H&E-stained images which are available and accessible in medical practice, making it feasible to evaluate CD276 expression for each patient with a pathological diagnosis. Besides, we investigated molecular biological interpretations of the pathomic model from a transcriptome perspective, thereby elucidating the potential mechanisms behind pathomic features.

Colon cancer is the leading cause of fatal tumors. Improving the prognosis of colon cancer patients remains a prominent issue in medical research. More accurate prognostic markers for colon cancer are being sought to guide clinical decision in precision medicine. It has been demonstrated that high expression of CD276 in tumors is associated with poor prognosis in a variety of human malignancies, including breast, colorectal, liver, and prostate cancers, and glioblastoma (33–38), and CD276 has become an attractive target for tumor immunotherapy (9, 39, 40), so we chose CD276 as an outcome indicator of COAD. Consistently, our study found that high expression of CD276 in COAD was associated with poor OS (**Figure 2B**). Univariate and multivariate analysis showed that CD276 was an independent risk factor for OS in COAD (**Figure 2C**). These results indicate that CD276 may be a prognostic indicator for COAD.

At present, the most commonly used methods for CD276 detection include immunohistochemistry, gene microarray, and next-generation sequencing analysis (41, 42), which are time-consuming and expensive, limiting extensive studies and applications of CD276. Histopathological images contain enormous information about tumor morphology and microenvironment, which plays a vital role in the prognosis of cancer patients (43–45). However, manual evaluation of H&E images cannot reliably conclude the expression of molecules, including CD276 and other biomarkers, those are critical to prognosis or treatment options. Machine learning models based on H&E-stained image features have been showed to be useful in some tumor classification and prognosis (46–48). Shamai et al. established a pathomic model to predict PD-1 expression in breast cancer (5), and Cao et al. conducted a pathomic model to predict microsatellite instability in colorectal cancer (49). In this study, the mRMR algorithm and the RFE algorithm were used to screen histopathological features, which are unable to identified by visual examination. Then GBM algorithm was used to construct a pathomic model, which showed AUC performance for the prediction of CD276 expression was 0.833 (95% CI: 0.784-0.882) in the training set and 0.758 (95% CI: 0.637-0.878) in the validation set, respectively (**Figures 3A, B**), suggesting good predictive performance of the model. In addition, we found that high PS was associated with poor prognosis (**Figure 4B**) and an independent risk factor for OS in COAD (**Figure 4C**), indicating that the pathomic model had predictive value for OS in COAD. This simple and convenient machine learning approach based on pathomics can be easily generalized to the prediction of other genes.

We explored the structural and functional alterations in pathomic features related to molecular behavior, providing corresponding biological support for the pathomic model. According to the GESA result, PS was implicated in several immune related pathways such as Cytokine-cytokine receptor interaction, interferon-gamma response, interferon-alpha response, inflammatory response, and Antigen processing and presentation (**Figures 5A, B**). Interferon-gamma is a vital cytokine in antitumor response. Dysregulated interferon-gamma response pathway inhibits immune checkpoint responses in tumor cells (50). Some inflammation and apoptosis related molecules, such as NF-kB1 and IL-1B, were significantly increased in PS-H group. NF-kB1 was reported to promote progression of COAD by driving metabolic and immune-related pathways (51). Elevated production of IL-1B is related to poor prognosis in a variety of malignancies, including colon cancer (52). Besides, our results indicated different abundances of immune cells in PS-H and PS-L groups. The above observations suggest a possible relationship between the pathomic model and immunity. The changes of gene composition and function involved in the pathomic model provide a theoretical basis for further hypothesis and experimental research.

While we are the first to construct a pathomic model to predict CD276 expression and present an integrated pathomic and transcriptomic approach to unveil potential biological mechanism underlying pathomic features, this study does have certain limitations. First and foremost, this research was carried out using data from the TCGA database. While the quality of TCGA data is commendable, it is important to acknowledge the possibility of patients with relatively incomplete clinical and pathological information, which could introduce potential bias into the research and analysis process. Secondly, it is essential to recognize that this study is a retrospective observational investigation, which inherently limits its ability to establish causality. Furthermore, due to constraints in available covariates for inclusion in the multivariate Cox regression analysis, it is possible that confounding factors might still influence the study’s results. Additionally, the absence of an external validation set reduces the variability of the data used in this study, failing to reflect the heterogeneity of COAD patients treated at different medical centers.

## 5 Conclusions

We constructed a pathomics-based machine learning model for CD276 prediction directly from H&E-stained images without additional genetic or immunohistochemical detections. Besides, this pathomic model may be an important prognostic biomarker for COAD patients.

## Data availability statement

The original contributions presented in the study are included in the article/**Supplementary Material**. Further inquiries can be directed to the corresponding authors.

## Ethics statement

This study is based on publicly available data, ensuring compliance with ethical standards and regulations.

## Author contributions

JL and CZ: Conception of the study. JL: conducted the study and collected dataset. CZ: performed bioinformatics and statistical analysis. JL: wrote the manuscript. DW and CZ: revised the manuscript. All authors reviewed the manuscript and approved the final version.
