# Supplementary figures and images for: Establishment of a pathomic-based machine learning model to predict CD276 (B7-H3) expression in colon cancer
Source: Front Oncol. 2024 Jan 8;13:1232192. doi: 10.3389/fonc.2023.1232192 (PMC10802857; doi:10.3389/fonc.2023.1232192)

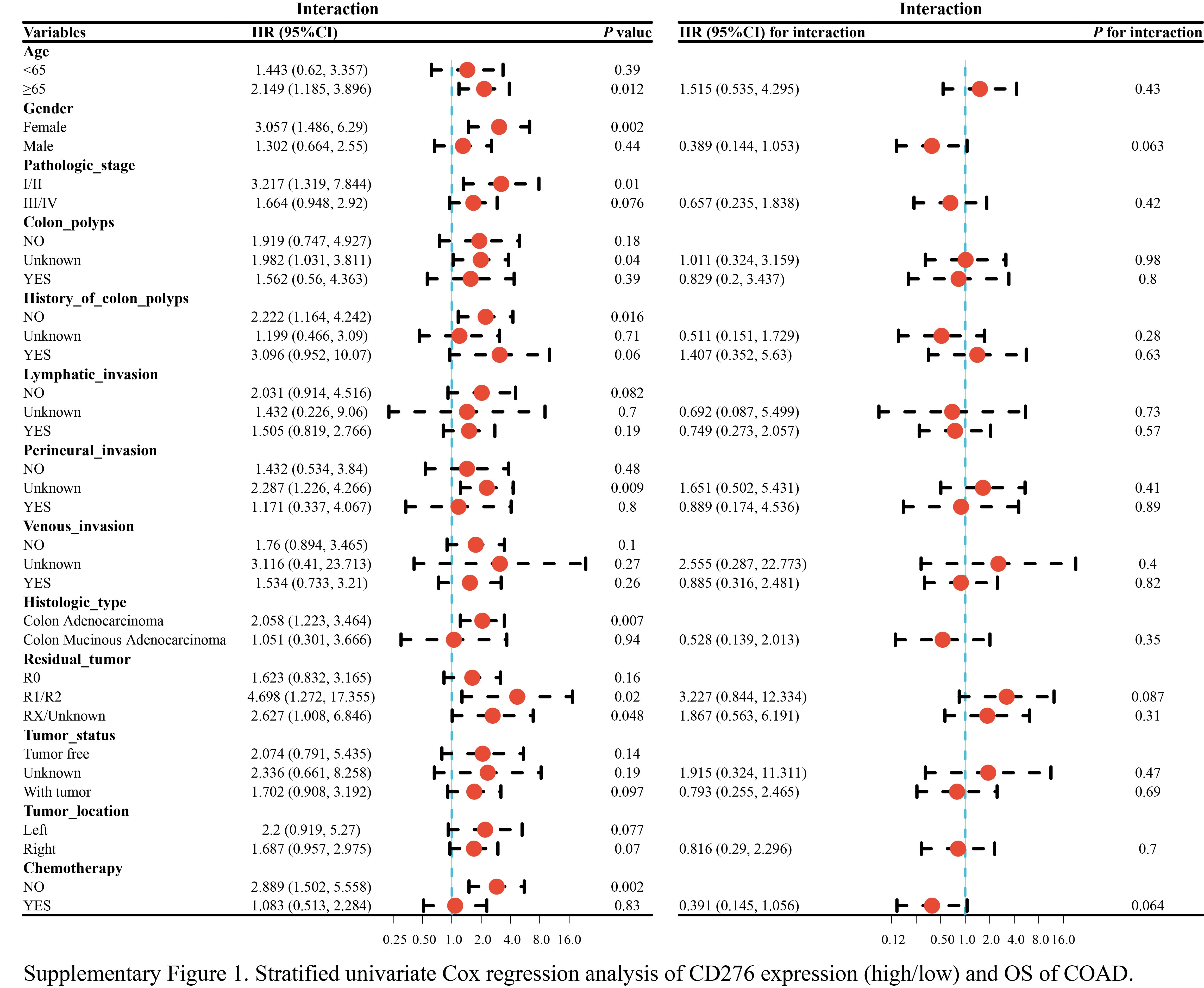

Supplement: Supplementary file 1 [file Image_1.jpeg]

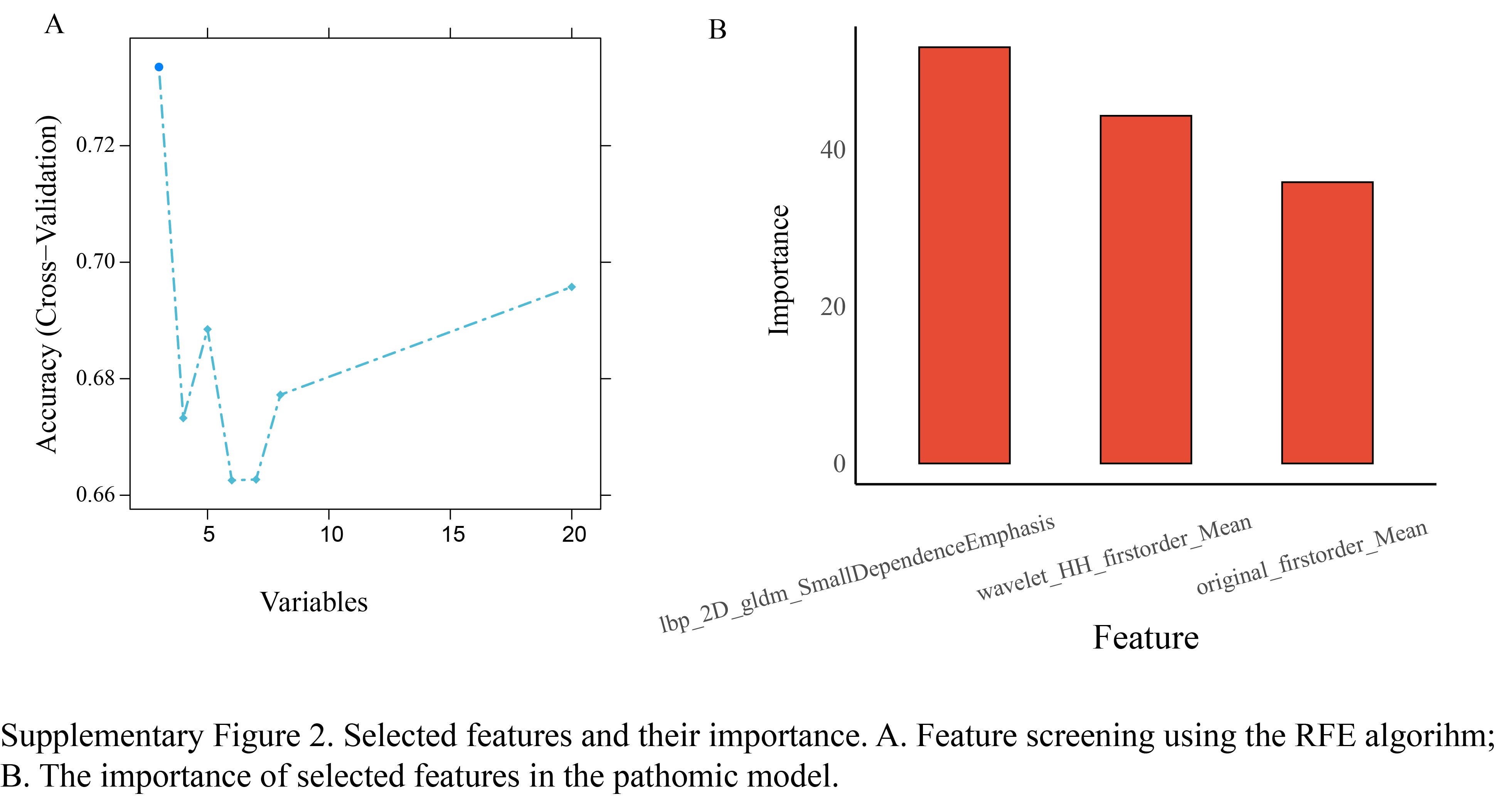

Supplement: Supplementary file 2 [file Image_2.jpeg]

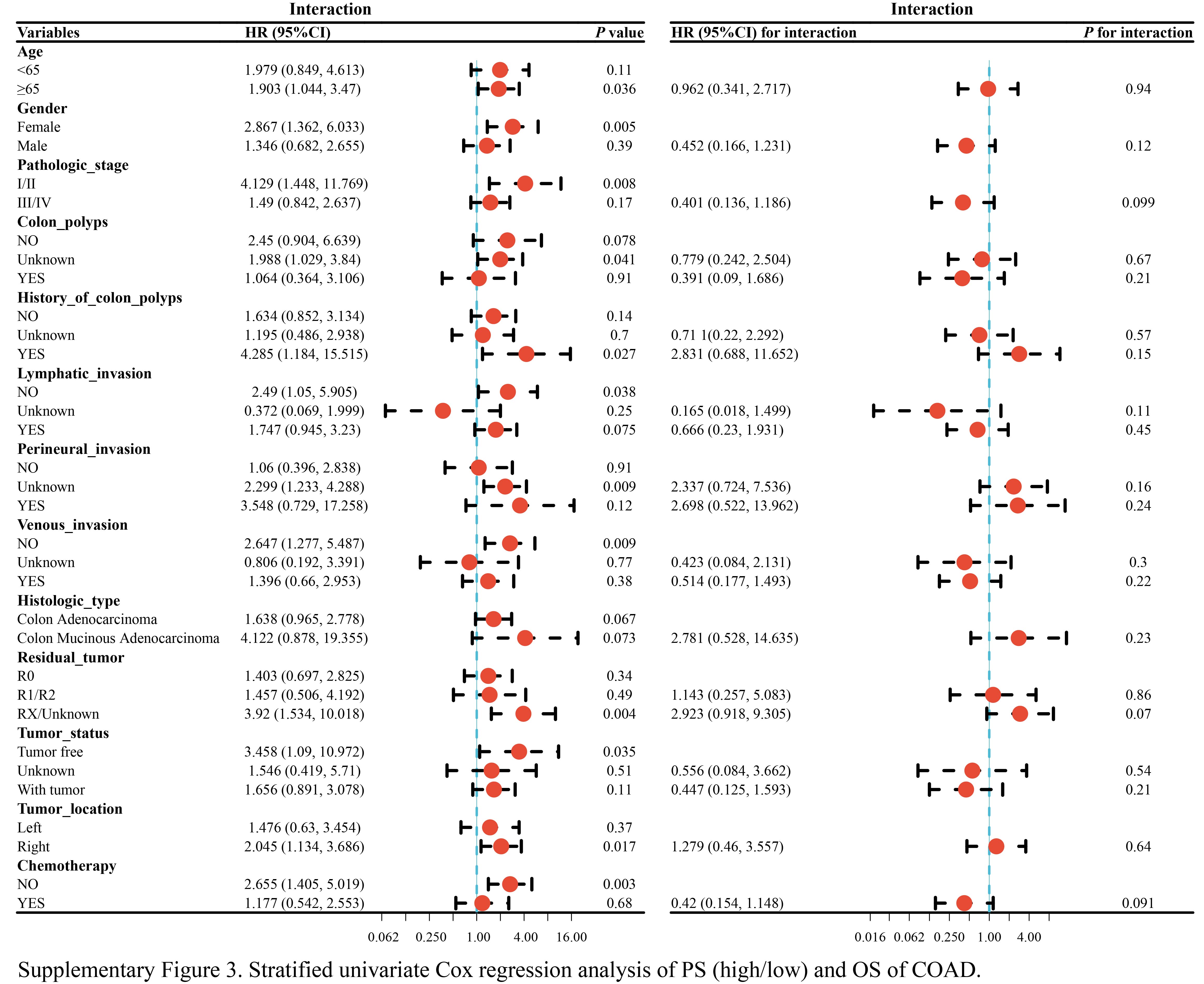

Supplement: Supplementary file 3 [file Image_3.jpeg]

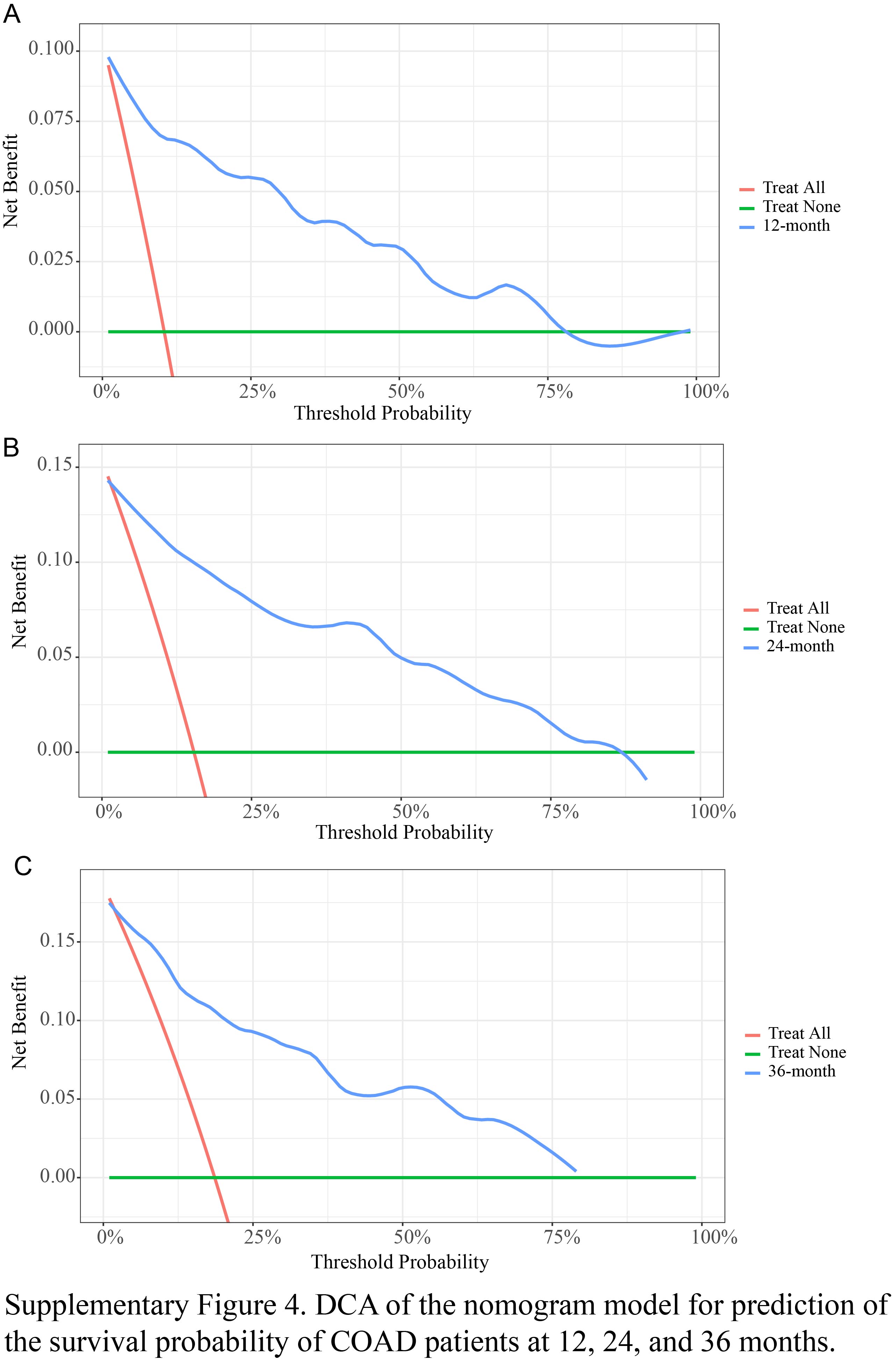

Supplement: Supplementary file 4 [file Image_4.jpeg]
