# Supplementary material for: Establishment of a pathomic-based machine learning model to predict CD276 (B7-H3) expression in colon cancer
Source: Front Oncol. 2024 Jan 8;13:1232192. doi: 10.3389/fonc.2023.1232192 (PMC10802857; doi:10.3389/fonc.2023.1232192)
Supplement: Supplementary file 5 [file Table_1.docx]

**Supplementary Table 1.** Characteristics of subjects in the PS-H and PS-L groups

| Characteristics | Total (n=332) | PS-L group (n=192) | PS-H group (n=140) | *P* value |
| --- | --- | --- | --- | --- |
| Age (years), n (%) |  |  |  | 0.971 |
| <65 | 132 (40) | 77 (40) | 55 (39) |  |
| ≥65 | 200 (60) | 115 (60) | 85 (61) |  |
| Gender, n (%) |  |  |  | 0.819 |
| Female | 160 (48) | 91 (47) | 69 (49) |  |
| Male | 172 (52) | 101 (53) | 71 (51) |  |
| Pathological stage, n (%) |  |  |  | 0.788 |
| I/II | 189 (57) | 111 (58) | 78 (56) |  |
| III/IV | 143 (43) | 81 (42) | 62 (44) |  |
| Colonic polyps, n (%) |  |  |  | 0.769 |
| No | 111 (33) | 65 (34) | 46 (33) |  |
| Unknown | 164 (49) | 92 (48) | 72 (51) |  |
| Yes | 57 (17) | 35 (18) | 22 (16) |  |
| History of colonic polyps, n (%) |  |  |  | 0.584 |
| No | 184 (55) | 107 (56) | 77 (55) |  |
| Unknown | 45 (14) | 23 (12) | 22 (16) |  |
| Yes | 103 (31) | 62 (32) | 41 (29) |  |
| Lymph node metastasis, n (%) |  |  |  | 0.79 |
| No | 180 (54) | 105 (55) | 75 (54) |  |
| Unknown | 22 (7) | 14 (7) | 8 (6) |  |
| Yes | 130 (39) | 73 (38) | 57 (41) |  |
| Perineural invasion, n (%) |  |  |  | 0.638 |
| No | 105 (32) | 62 (32) | 43 (31) |  |
| Unknown | 195 (59) | 114 (59) | 81 (58) |  |
| Yes | 32 (10) | 16 (8) | 16 (11) |  |
| Venous invasion, n (%) |  |  |  | 0.957 |
| No | 220 (66) | 128 (67) | 92 (66) |  |
| Unknown | 36 (11) | 20 (10) | 16 (11) |  |
| Yes | 76 (22) | 44 (23) | 32 (23) |  |
| Pathological type, n (%) |  |  |  | 0.07 |
| Colon adenocarcinoma | 285 (86) | 171 (89) | 114 (81) |  |
| Colon mucinous adenocarcinoma | 47 (14) | 21 (11) | 26 (19) |  |
| Residual tumor, n (%) |  |  |  | 0.468 |
| R0 | 254 (77) | 148 (77) | 106 (76) |  |
| R1/R2 | 20 (6) | 9 (5) | 11 (8) |  |
| Rx/Unkown | 58 (17) | 35 (18) | 23 (16) |  |
| Tumor status, n (%) |  |  |  | 0.52 |
| Tumor free | 153 (46) | 88 (46) | 65 (46) |  |
| Unknown | 28 (8) | 19 (10) | 9 (6) |  |
| With tumor | 151 (45) | 85 (44) | 65 (47) |  |
| Tumor location, n (%) |  |  |  | 0.895 |
| Left | 133 (40) | 78 (41) | 55 (39) |  |
| Right | 199 (60) | 114 (59) | 85 (51) |  |
| Chemotherapy, n (%) |  |  |  | 0.808 |
| No | 210 (63) | 123 (64) | 87 (62) |  |
| Yes | 122 (37) | 69 (36) | 53 (38) |  |
